# Respiratory Burst Oxidase Homolog Gene A Is Crucial for *Rhizobium* Infection and Nodule Maturation and Function in Common Bean

**DOI:** 10.3389/fpls.2017.02003

**Published:** 2017-11-23

**Authors:** Manoj-Kumar Arthikala, Jesús Montiel, Rosana Sánchez-López, Noreide Nava, Luis Cárdenas, Carmen Quinto

**Affiliations:** ^1^Ciencias Agrogenómicas, Escuela Nacional de Estudios Superiores Unidad León, Universidad Nacional Autónoma de México, León, Mexico; ^2^Instituto Nacional de Investigación y Tecnología Agraria y Alimentaria, Centro de Biotecnología y Genómica de Plantas, Universidad Politécnica de Madrid, Madrid, Spain; ^3^Departamento de Biología Molecular de Plantas, Instituto de Biotecnología, Universidad Nacional Autónoma de México, Cuernavaca, Mexico

**Keywords:** infection thread, NADPH oxidase homologs, nodule, *Phaseolus vulgaris* symbiosis, *Rhizobium tropici*, ROS

## Abstract

Reactive oxygen species (ROS) produced by respiratory burst oxidase homologs (RBOHs) regulate numerous plant cell processes, including the symbiosis between legumes and nitrogen-fixing bacteria. Rapid and transient ROS production was reported after *Phaseolus vulgaris* root hairs were treated with Nod factors, indicating the presence of a ROS-associated molecular signature in the symbiosis signaling pathway. *Rboh* is a multigene family containing nine members (*RbohA–I*) in *P. vulgaris*. RNA interference of *RbohB* suppresses ROS production and attenuates rhizobial infection thread (IT) progression in *P. vulgaris* root hairs. However, the roles of other *Rboh* members in symbiotic interactions are largely unknown. In this study, we characterized the role of the NADPH oxidase-encoding gene *RbohA* (Phvulv091020621) in the *P. vulgaris*–*Rhizobium tropici* symbiosis. The spatiotemporal activity of the *RbohA* promoter colocalized with growing ITs and was associated with vascular bundles in developing nodules. Subcellular localization studies indicated that RBOHA was localized in the plasma membrane of *P. vulgaris* root hairs. After rhizobial inoculation, PvRBOHA was mainly distributed in the infection pocket and, to a lesser extent, throughout the IT. In *PvRbohA* RNAi lines, the rhizobial infection events were significantly reduced and, in successful infections, IT progression was arrested within the root hair, but did not impede cortical cell division. *PvRbohA*-RNAi nodules failed to fix nitrogen, since the infected cells in the few nodules formed were empty. *RbohA*-dependent ROS production and upregulation of several antioxidant enzymes was attenuated in rhizobia-inoculated *PvRbohA*-RNAi roots. These combined results indicate that *PvRbohA* is crucial for effective *Rhizobium* infection and its release into the nodule cells. This oxidase is partially or indirectly required to promote nodule organogenesis, altering the expression of auxin- and cyclin-related genes and genes involved in cell growth and division.

## Introduction

Reactive oxygen species (ROS) are important signals that regulate numerous biological processes in living cells. Several enzymes have been implicated in ROS generation. The most important and well-characterized ROS-generating system is the NADPH-dependent oxidase (Nox) complex. Nox members have been identified and characterized in fungi, plants, and animals (Lambeth, 2004). Nox are integral membrane proteins that catalyze the production of superoxide anion by reducing molecular oxygen using NADPH as the electron donor (Umeki, 1994). In plants, NADPH oxidases belong to the multigene respiratory burst oxidase homolog (RBOH) family, which contains up to 10 different members in the model plant *Arabidopsis thaliana* (Chang et al., 2016; Kaur and Pati, 2016). RBOHs possess six transmembrane regions, two heme groups, and cytosolic FAD- and NADPH-binding domains in the carboxy terminus (Suzuki et al., 2011). The N-terminal region contains two Ca^2+^-regulated domains including EF-hand motifs and specific phosphorylation sites targeted by Ca^2+^-regulated protein kinases (Ogasawara et al., 2008). Several recent studies reported that RBOHs participate in signaling pathways associated with cell elongation, hormonal signaling, root hair growth, pollen–stigma interactions, plant–pathogen interactions, and plant–symbiont interactions (see review Singh et al., 2016).

In legumes, RBOH-dependent ROS production has been implicated in the establishment of symbiotic associations with *Rhizobium* (reviewed in Montiel et al., 2016a). Our group described a fast and transient ROS burst in *Phaseolus vulgaris* root hair cells treated with Nod factors (NFs), which appears within seconds of treatment and is maintained for approximately 3 min; this is one of the fastest responses reported in the symbiosis signaling pathway (Cárdenas et al., 2008). Treatment of root hair cells with the NADPH-oxidase inhibitor diphenyleneiodonium suppressed the NF-induced ROS burst (Cárdenas et al., 2008) and prevented nodule formation in rhizobial-inoculated legume roots (Peleg-Grossman et al., 2007), suggesting that RBOH mediates ROS generation in the legume–*Rhizobium* symbiosis. During rhizobial infection, superoxide anions accumulate to high levels in the infection thread (IT) and nodule primordia of *Medicago sativa* roots (Santos et al., 2001). ROS accumulated in intercellular infection pockets that precede stem nodulation in *Sesbania rostrata* has been shown to be required for initiation of nodulation of hydroponic roots of *S. rostrata* (D’haeze et al., 2003). Loss-of-function analysis of ROP9-GTPase, which regulates *MtRbohE*/*3* expression in *Medicago truncatula*, blocked rhizobial IT formation in root hair cells (Kiirika et al., 2012). A recent report indicated that *MtRbohE* is activated in arbusculated cells and is involved in root cortex colonization; however, *MtRbohE-*RNAi plants were not affected in nodule formation (Belmondo et al., 2016). Silencing of *MtRbohA* does not affect nodule development, but does affect nitrogen fixation capacity in *M. truncatula* (Marino et al., 2011). In *P. vulgaris*, the *Rboh* gene family is composed of nine members and analysis of their expression profiles indicated that four of these (*RbohA–D*) were abundant in several organs including nodules. Silencing of *RbohB* blocked IT formation and affected nodule development and function (Montiel et al., 2012), whereas *RbohB* overexpression increased the number of infection events and nodule numbers and enhanced the level of fixed nitrogen (Arthikala et al., 2014). These previous studies showed that RBOH-mediated ROS production is essential for proper growth of rhizobial ITs, nodule organogenesis, and nodule function. However, several reports support the relevance of studying the different RBOH members, as each member can have distinct roles in the same biological process, ranging from synergistic to non-redundant (Kaur et al., 2014). To gain insight into the interplay among RBOH members and into their potential different roles in the legume–rhizobium symbiosis, we investigated *PvRbohA* (Phvulv091020621), which was expressed at higher levels in nodules than in other organs (Montiel et al., 2012). We found that PvRBOHA sustains rhizobial invasion and nodule formation. We link PvRBOHA to ROS production, and show that it participates in IT progression, nodule maturation, and nodule function during *Rhizobium* symbiotic interactions.

## Materials and Methods

### Plant Material, Growth Conditions, Rhizobial Infection, and Root Hair Isolation

*Phaseolus vulgaris* cv. Negro Jamapa seeds were used for this study. Surface-sterilized seeds were germinated for 2 days in darkness at 28°C. Then, 2-day-old seedlings were planted in pots containing sterile vermiculite, inoculated or not with *Rhizobium tropici* CIAT899 strain (OD_600_ = 0.05), and irrigated regularly with Broughton and Dilworth (1971) (B&D) medium without nitrate (KNO_3_). Only the crown root nodulation zone was collected at different time points. Nodules were individually collected at 14, 28, and 33 days post-inoculation (dpi), frozen immediately in liquid nitrogen, and stored at -80°C. *Rhizobium*-treated root hairs were isolated as described previously (Wan et al., 2005). Briefly, surface-sterilized *P. vulgaris* seeds were sown on agar plates containing 1× B&D medium. An *R. tropici* bacterial suspension was inoculated onto the roots of 2-day-old seedlings using a mist sprayer, and the seedlings were incubated at 28°C for 3 days. Control seedlings were mist-sprayed with water. Subsequently, whole roots were detached from the shoots, frozen in liquid nitrogen, and stored immediately at -80°C; this material was used to isolate root hairs.

### Plasmid Construction and Hairy Root Transformation

The *PvRbohA* promoter upstream of the *PvRbohA* translation start site was obtained by amplifying 3,312 bp of the promoter sequence from *P. vulgaris* genomic DNA using primer-specific oligonucleotides (Supplementary Table S1), and the amplified fragment was cloned into the pENTR/SD/D-TOPO vector (Invitrogen, United States). The Gateway LR reaction was performed between the entry vector pENTR/SD/D-TOPO-*pPvRbohA* and the destination vector pBGWSF7.0 (Karimi et al., 2002) according to the manufacturer’s instructions (Invitrogen, United States).

Next, the PvRBOHA protein construct was generated by amplifying the *PvRbohA* coding sequence from *P. vulgaris* cDNA using the appropriate oligonucleotides (Supplementary Table S1), and the coding fragment was cloned into the pENTR/SD/D-TOPO vector (Invitrogen, United States). The Gateway LR reaction was performed between the entry vector pENTR/SD/D-TOPO-*PvRbohA* and the destination vector pEARLEY104 according to the manufacturer’s instructions (Invitrogen, United States). The resulting binary vector generates a translational N-terminal fusion of PvRBOHA and yellow fluorescent protein (YFP). The pEARLEY104 vector expressing YFP was used as a control.

To generate the *RNAi* construct, a 217-bp fragment corresponding to the 3′-untranslated region of *PvRbohA* (Phvulv091020621) was amplified from *P. vulgaris* root cDNA using the appropriate oligonucleotides (Supplementary Table S1), and the amplified product was cloned into the pTdT-DC-RNAi vector (Valdés-López et al., 2008). A control plasmid was generated in a similar manner by inserting a truncated sequence lacking the target sequence (from *A. thaliana* pre-mir159) as described in our previous work (Montiel et al., 2012).

The correct orientation of all constructs was confirmed by sequencing. The recombinant plasmids were introduced into *Agrobacterium rhizogenes* strain K599 and then transformed into *P. vulgaris* roots using the hairy root transformation method described previously (Estrada-Navarrete et al., 2007).

### Microscopy

Transgenic hairy roots expressing the *PvRbohA::GFP-GUS* promoter, 35S:*YFP-RBOHA*, and 35S:*PvRbohA*-RNAi vectors were selected by monitoring the fluorescence of green fluorescent protein (GFP), YFP, and red fluorescent protein (RFP), respectively, using an epifluorescence stereomicroscope (SZX7, Olympus, Japan). Bright-field and fluorescence microscopy were performed with an Axioskop microscope (Zeiss) or with an LSM-510 META confocal laser-scanning microscope (Zeiss). The confocal images (single planes or a *Z*-projection series composed of 15–20 images taken at 1.2 μm increments) were processed using LSM 5 software. GFP was excited with an argon laser (488 nm) and the emitted fluorescence from 510 to 540 nm was collected. YFP was excited with an argon laser (513 nm) and the emitted fluorescence from 527 nm was collected. RFP was excited with a solid-state laser (561 nm) and the emitted fluorescence was filtered using a 640-/650-nm bandpass filter. GUS activity was detected according to a previously published method (Jefferson, 1987).

### RNA Isolation and RT-qPCR Analysis

Plant tissues were ground in liquid N_2_ and total RNA was extracted using TriPure Isolation Reagent (Roche, Mannheim, Germany) according to the manufacturer’s instructions. Contaminant genomic DNA was eliminated by incubating the RNA samples for 15 min at 37°C with RNase-free DNase (1 U μL^-1^). RNA integrity was determined by electrophoresis, and RNA concentration was determined using an ND-2000 spectrophotometer (Nanodrop, Thermo Fisher Scientific, Wilmington, DE, United States). Quantitative real-time PCR was performed using the iScript^TM^ One-Step RT-PCR Kit with SYBR^®^ Green and an iQ5 Multicolor Real-time PCR Detection System according to the manufacturer’s instructions (Bio-Rad, CA, United States). Each reaction contained 40 ng of RNA as template. A control sample lacking reverse transcriptase (RT) was included to confirm the absence of contaminant DNA. Relative gene expression levels were calculated using the 2^-Δ*CT*^ method, with ΔCT = CT_gene_ - CT_reference gene_. The *P. vulgaris* reference genes *EF1*α and *IDE* were used as internal controls as described previously (Islas-Flores et al., 2011; Borges et al., 2012). The relative expression values were normalized with respect to the expression levels of these two reference genes, which were calculated according to the method of Vandesompele et al. (2002). Reported values are averages of two or three biological replicates, and each sample was assessed in triplicate. Expression levels of the above-mentioned genes were quantified using gene-specific oligonucleotides as listed in Supplementary Table S1.

### ROS Determination

Composite plants grown in glass tubes (15 cm) containing B&D medium were used to determine O_2_^-^ levels in transgenic roots at 10 days post-emergence (dpe). *In situ* O_2_^-^ was estimated using the nitroblue tetrazolium (NBT) staining method as described by Montiel et al. (2012). Samples were incubated for 1 h in darkness at room temperature, and then roots were cleared in 90% ethanol. In the presence of O_2_^-^, NBT forms an insoluble blue formazan precipitate. Superoxide was quantified as described previously by Ramel et al. (2009). To determine the formazan content in NBT-stained roots, tissue was ground briefly in liquid N_2_, solubilized in 2 M KOH-DMSO (1:1.16, v/v), and then samples were centrifuged for 10 min at 12,000 *g*. The optical density at A_630_ was immediately measured and compared with a standard curve obtained from known amounts of NBT in 2 M KOH-DMSO.

### Acetylene Reduction

Nitrogenase activity in transgenic nodules was determined at 21 and 28 dpi by measuring acetylene reduction. Nodulated roots of composite plants at 21 and 28 dpi were incubated in acetylene gas for 30 min, and ethylene production was determined by gas chromatography (Variant model 3300) as described previously by Ramírez et al. (1999). Nitrogenase-specific activity was expressed as μmol^-1^ of C_2_H_2_ h^-1^ g^-1^ of nodule dry weight.

### Rhizobial Infection Phenotype and Nodule Histology

To analyze the rhizobial infection phenotype, transgenic roots inoculated with *R. tropici-GUS* were harvested at 7 dpi and stained for GUS activity (Jefferson, 1987). Control and *PvRbohA*-RNAi roots were examined for IT status with an Axioskop light microscope. For histological examination, nodules were processed in a mixture of 2.5% glutaraldehyde and 4% paraformaldehyde in 0.1 M Na-cacodylate buffer (pH 7.2), post-fixed with 1% osmium tetroxide, and dehydrated using an ethanol series (10–100%) as described by Sánchez-López et al. (2011). Then, samples were embedded in LR White resin. Semi-thin sections (0.5–1.0 mm) were prepared using an ultramicrotome (Ultracut, Leica) and stained with 0.1% toluidine blue. The stained tissues were examined with a bright-field microscope (DMLB, Leica).

### Rhizobia Reisolation Assay

Nodules were isolated from transgenic control and *PvRbohA*-RNAi roots at 21 dpi. These nodules were surface-sterilized by immersion in absolute ethanol for 30 s and 10% sodium hypochlorite for 10 min, followed by three washes with sterile distilled water. Each nodule was then homogenized in five volumes of 100 mM MgCl_2_ using an Eppendorf micropestle. The homogenate was serial diluted (10^0^–10^-9^) with 100 mM MgCl_2_, spread (100 μL) on PY medium (5 g peptone and 3 g yeast extract per liter) supplemented with 20 μg mL^-1^ nalidixic acid (Sigma), and incubated at 30°C. Colonies were counted after 24 h.

### Statistical Analysis

Statistical analyses were computed using GraphPad Prism version 5.00 for Windows (GraphPad Software, San Diego, CA, United States). Significance tests were performed using an unpaired Student’s *t*-test or one-way ANOVA and Tukey’s multiple comparison test. Differences were considered significant if *P* < 0.05. Results are presented as means ± standard error of the mean.

## Results

### *PvRbohA* Expression Pattern in Response to Rhizobial Inoculation

**FIGURE 1:**
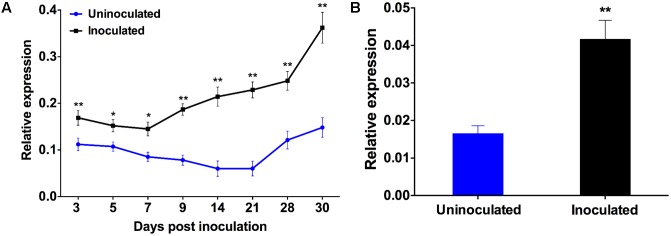
Quantitative real-time PCR analysis of *PvRbohA*. Temporal profiles of *RbohA* expression were determined in **(A)** roots or **(B)** root hairs cells at different time intervals and 3 days post-inoculation (dpi) with *R. tropici*, respectively. Transcript accumulation was normalized to the expression of the *EF1*α and *IDE* reference genes. The reported values represent three biological replicates (**A**; *n* > 9) or two biological replicates (**B**; *n* > 6). The statistical significance of differences between uninoculated and *R. tropici*-inoculated samples was determined using an unpaired two-tailed Student’s *t*-test (^∗^*P* < 0.05; ^∗∗^*P* < 0.01). Error bars represent means ± SEM.

Our previous work showed that *PvRbohB* has a crucial role during IT progression and nodule development in *P. vulgaris* (Montiel et al., 2012). Here, we assessed the function of *PvRbohA* in the symbiotic interaction between *P. vulgaris* and *R. tropici*. First, we investigated the *PvRbohA* expression pattern in *P. vulgaris* roots inoculated with *R. tropici* by monitoring *PvRbohA* transcript levels using RT-qPCR at different dpi, i.e., 3, 5, 7, and 9 dpi (early stages of symbiosis) in inoculated roots and 14, 21, 28, and 30 dpi (late stages of symbiosis) in nodulated roots (**Figure 1A**). The *PvRbohA* transcript abundance was higher during the late stages (14, 21, and 28 dpi) than during the early stages (3, 5, 7, and 9 dpi). *PvRbohA* transcript levels drastically increased at the onset of nodule senescence (30 dpi; **Figure 1A**). In general, *PvRbohA* expression levels were higher in *Rhizobium*-inoculated roots than in uninoculated wild-type roots (**Figure 1A**).

We also measured *PvRbohA* transcript accumulation in 14, 21, 28, and 33 dpi wild-type nodules. The relative *PvRbohA* expression levels increased dramatically in senesced non-nitrogen-fixing nodules at 28 and 33 dpi (i.e., those that were green due to leghemoglobin degradation) compared with the levels in active nitrogen-fixing nodules at 14 and 21 dpi (i.e., those that were pink due to the presence of leghemoglobin, which is required for oxygen-sensitive nitrogenase activity; Supplementary Figure S1).

Considering that rhizobial root infection generally occurs via root hair cells, we quantified the *PvRbohA* transcript abundance in *P. vulgaris* root hairs. Root hairs were isolated at 3 dpi, and transcript levels were measured by RT-qPCR. *PvRbohA* transcript levels significantly increased in root hair cells at 3 dpi (**Figure 1B**). These combined results suggest that *Rhizobium* infection significantly enhances *PvRbohA* gene expression at different stages of nodulation.

### Subcellular Localization of PvRBOHA

**FIGURE 2:**
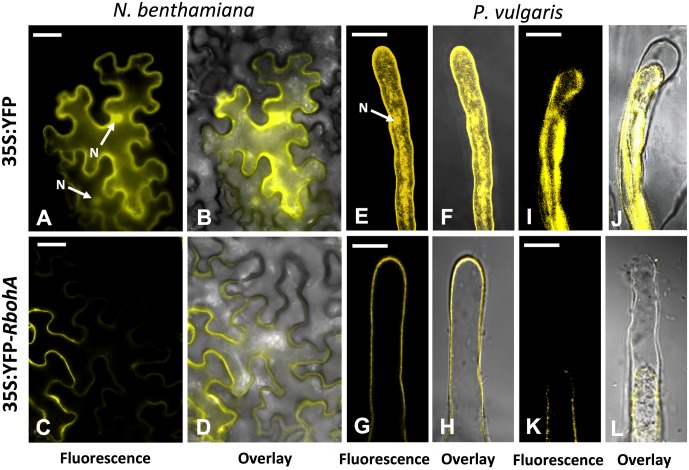
Subcellular localization of PvRBOHA. The *PvRbohA* coding region was cloned in pEarleyGate104 to construct an N-terminal YFP fusion, and introduced into *N. benthamiana* leaf cells and *P. vulgaris* hairy roots to determine the subcellular localization. Images were obtained with a confocal microscope. **(A,B)** Non-fused 35S-YFP (control). **(C,D)** The YFP-PvRBOHA construct exhibits plasma membrane localization in *N. benthamiana* leaf cells. **(E,F)** Images of growing root hairs from control plants, and **(G,H)** YFP-PvRBOHA in *P. vulgaris* hairy roots. Plasmolysis was induced in *P. vulgaris* root hair cells by treatment with 200 mM NaCl for 10 min before imaging; **(I,J)** control, and **(K,L)** YFP-PvRBOHA. Overlay: transmitted light and yellow fluorescence. Bars = 20 μm. N, nucleus.

*PvRbohA* encodes a predicted membrane protein of 876 aa (www.psort.org). To corroborate the *in silico* prediction of *PvRbohA*, its coding region was fused to the YFP in the N-terminus under the transcriptional regulation of the 35S promoter and transgenically expressed in *Nicotiana benthamiana* leaves by agroinfiltration with *Agrobacterium tumefaciens*, or in transgenic *P. vulgaris* hairy roots by infection with *A. rhizogenes*. Transgenic leaves or hairy roots expressing non-fused YFP served as controls. As anticipated, the non-fused YFP was observed in both the cytoplasm and nuclei of *N. benthamiana* leaf cells (**Figures 2A,B**), *P. vulgaris* hairy root cells (Supplementary Figure S2A), and root hair cells (**Figures 2E,F**). Yellow fluorescence (YFP-PvRBOHA) was detected in the plasma membrane of all tested tissues including *N. benthamiana* leaves (**Figures 2C,D**), transgenic hairy roots of *P. vulgaris* (Supplementary Figure S2B), and root hair cells of *P. vulgaris* (**Figures 2G,H**). Root hairs expressing YFP-PvRBOHA were treated with 200 mM NaCl to induce plasmolysis, and fluorescence remained associated with the plasma membrane after retraction from the cell wall (**Figures 2K,L**). By contrast, plasmolysis of control root hairs showed that YFP fluorescence remained in the cytoplasm (**Figures 2I,J**). These subcellular PvRBOHA localization results are consistent with the plasma membrane localization of RBOHs in other cell types (Suzuki et al., 2011).

Recently, our group described that PvRBOHB is located in the infection pocket and the growth pole of the IT in *P. vulgaris* root hairs (Montiel et al., 2016a). Similarly, in this study, the subcellular distribution of YFP-PvRBOHA was visualized in rhizobia-inoculated roots by confocal microscopy. A particularly intense signal was detected in the infection pocket; however, weaker fluorescence was also observed that could be associated with the IT membrane and the base of the root hair cell (Supplementary Figures S2C,D). This distribution pattern of PvRBOHA suggests that it has a well-defined role during IT progression.

### Spatiotemporal Expression of *PvRbohA*

**FIGURE 3:**
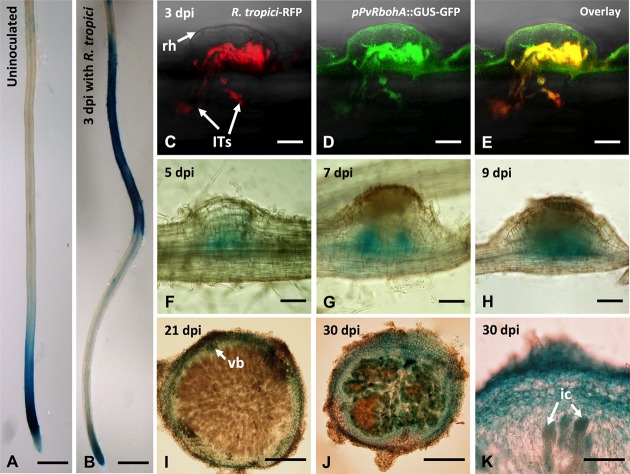
Promoter analysis of *PvRbohA* in transgenic *P. vulgaris* roots and nodules. Spatiotemporal pattern of *PvRbohA* expression revealed by a promoter::GUS-GFP construct in 10-day-old transgenic hairy roots incubated with GUS substrate. **(A)** Uninoculated root. **(B)** *R. tropici*-inoculated root at 3 dpi. Confocal microscopy imaging of *PvRbohA* promoter activity in *Rhizobium*-infected root hair cell and in growing ITs at 3 dpi. **(C)** Red fluorescence emitted by *R. tropici* CIAT899 Ds-Red. **(D)** *PvRbohA* expression revealed by promoter::GUS-GFP and **(E)** overlay. *PvRbohA* promoter activity in the developing nodules at **(F)** 5 dpi, **(G)** 7 dpi, and **(H)** 9 dpi. **(I)** Mature 21-dpi nodule section showing *PvRbohA* promoter activity restricted to the nodule cortex and vascular bundles (vb). **(J)** With the onset of nodule senescence (30 dpi), the promoter was active in the central tissue containing infected cells. **(K)** Higher magnification of a senescing nodule section showing promoter::GUS-GFP activity in infected cells. rh, root hair; ITs, infection threads; ic, infected cell; hpi, hours post-inoculation; dpi, days post-inoculation. Bars: **(A,B)** 1 mm; **(C–E)** 10 μm; **(F,G)** 100 μm; **(H,I)** 200 μm; **(J)** 150 μm; **(K)** 50 μm.

We assessed the spatiotemporal activity of the *PvRbohA* promoter during rhizobial symbiosis. We cloned a 3.4-kb fragment of the *PvRbohA* promoter (*pPvRbohA*) that was immediately upstream of the *PvRbohA* translation initiation codon and generated transcriptional fusions to the GUS-GFP coding sequences. The *pPvRbohA*::GUS-GFP construct was transfected into *P. vulgaris* using the hairy root transformation method, and transgenic roots were then inoculated with *R. tropici*. Strong GUS staining was observed in the subapical region of uninoculated transgenic root tips (**Figure 3A**). Next, we analyzed the *PvRbohA* promoter activity in transgenic roots at 3 dpi with *R. tropici*. GUS activity was observed in the root differentiation and maturation zones, confirming that the *PvRbohA* promoter was induced by rhizobial infection (**Figure 3B** and Supplementary Figure S3A). GUS staining also was observed in root hair cells in the root differentiation and maturation zones (Supplementary Figure S3B). By contrast, GUS staining was not observed in mock-treated transgenic roots or in *R. tropici*-inoculated roots transformed with the GUS-GFP vector lacking the *PvRbohA* promoter (data not shown).

To investigate *pPvRbohA*::GUS-GFP activity during rhizobial invasion, transgenic roots were inoculated with *R. tropici* expressing a fluorescent Ds-Red marker, and the promoter activity was determined using confocal microscopy. Roots that had been inoculated (3 dpi) displayed GFP fluorescence in the *Rhizobium*-infected root hair cell (**Figures 3C–E**). The *pPvRbohA* promoter was also active during nodule organogenesis (starting at 5 dpi), with intense GUS staining in the emerging vascular bundles of the nodule primordium (**Figures 3F–H**). In the mature nodule, *pPvRbohA* promoter activity was detected in the cortex and vascular bundles, but not in infected cells in the central nodule zone that contained nitrogen-fixing bacteroids (**Figure 3I**). By contrast, GUS staining was observed in the cortex and central zone containing infected cells during nodule senescence (**Figures 3J,K**). This was confirmed by sectioning these GUS-stained nodules. These spatiotemporal expression patterns of the *PvRbohA* promoter suggest that *RbohA* participates in rhizobial invasion of root hairs, IT progression, nodule organogenesis, and nodule senescence.

### Downregulation of *RbohA* Expression in *P. vulgaris* Composite Plants

**FIGURE 4:**
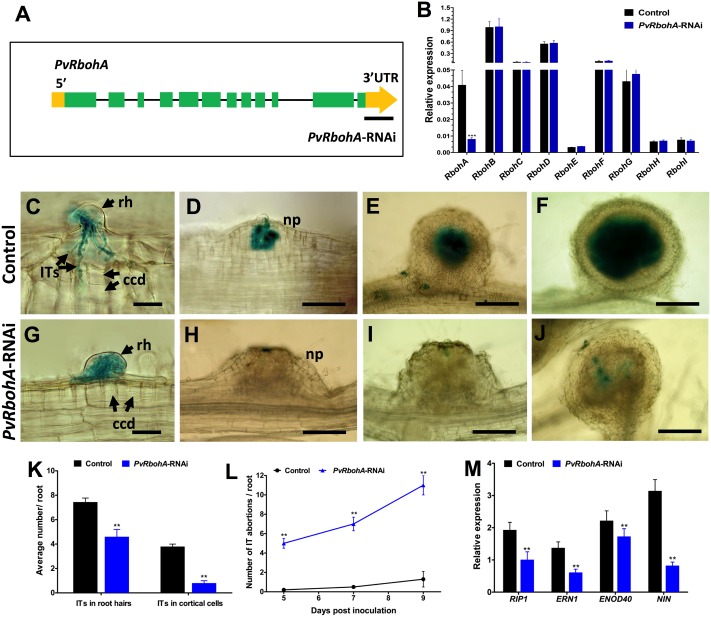
qPCR analysis of *Rboh* gene family transcripts, infection events, phenotype, and expression profile of early nodulin genes in *PvRbohA*-RNAi roots. **(A)** Illustration of the *PvRbohA* gene structure predicted using the GeneWise DNA search tool (http://www.ebi.ac.uk/Tools/psa/genewise/). Green boxes indicate exons, gray lines indicate introns, and yellow bars indicate UTRs. The black line designates the *PvRbohA* sequence used for silencing the target gene. **(B)** *P. vulgaris* transgenic hairy roots expressing the *PvRbohA*-RNAi construct were analyzed at 10 days post-emergence (dpe) to measure the transcript abundance of the nine *Rboh* family genes. Transcript accumulation was normalized to the expression of the *EF1*α and *IDE* reference genes. RT-qPCR data are the averages of three biological replicates (*n* > 9). The statistical significance of differences between control and *PvRbohA*-RNAi root samples was determined using an unpaired two-tailed Student’s *t*-test (^∗∗∗^*P* < 0.001). Error bars represent means ± SEM. Transgenic hairy roots were inoculated with *R. tropici* expressing a β-glucuronidase (GUS) marker. Roots were stained for GUS and observed using a light microscope **(C–J)** at 3 **(C,G)**, 5 **(D,H)**, 14 **(E,I)**, and 21 dpi **(F,J)**. **(C)** Control root showing typical IT progression, with branching and outer cortical cell divisions. **(D)** Nodule primordium colonized with rhizobia. Representative **(E)** young and **(F)** mature nodule images showing fully colonized central tissues. By contrast, *PvRbohA*-RNAi roots show **(G)** a thick IT arrested within the root hair cell, **(H)** nodule primordium, and **(I)** young nodule devoid of rhizobial colonization. **(J)** Few mature *PvRbohA*-RNAi nodules were sparingly colonized with rhizobia. **(K)** Quantitative data showing the average number of ITs observed in root hair cells and dividing cortical cells at 7 dpi **(L)** IT progression and inhibition in control and *PvRbohA*-RNAi roots. **(M)** Quantitative RT-PCR analysis showing expression (relative to uninoculated transgenic roots) of early nodulins such as *PvRIP1* and *PvERN1* at 24 hpi, and *PvENOD40* and *PvNIN* at 72 hpi. The values represent averages of three biological replicates [*n* > 36 for **(K,L)** and *n* > 9 for **(M)**]. The statistical significance of differences between control and *PvRbohA*-RNAi roots was determined using an unpaired two-tailed Student’s *t*-test (^∗∗^*P* < 0.01). Error bars represent means ± SEM. rh, root hair; ccd, cortical cell division; np, nodule primordium; IT, infection thread. Bars: **(C,G)** 20 μm; **(D,H)** 50 μm; **(E,I)** 100 μm; **(F,J)** 200 μm.

To functionally characterize *RbohA* during root nodule symbiosis, *P. vulgaris* transgenic roots were generated by *A. rhizogenes* that expressed an RNAi construct to specifically silence *PvRbohA* (henceforth *PvRbohA*-RNAi; **Figure 4A**) and as control, an RNAi lacking the target sequence (henceforth “control roots”). An RT-qPCR analysis of 10 dpe hairy roots confirmed the reduction of *RbohA* mRNA levels, with levels of around 80% in transgenic roots expressing *PvRbohA*-RNAi compared with the levels in transgenic control roots (**Figure 4B**). We also measured the transcript levels of the other eight *PvRboh* genes using RT-qPCR analysis, which indicated that the *PvRbohA*-RNAi construct specifically downregulated *PvRbohA* transcripts in transgenic roots, whereas the levels of the other *PvRboh* transcripts were not affected (**Figure 4B**). These combined results indicate that the *PvRbohA*-RNAi construct specifically downregulated *RbohA* transcript levels in *P. vulgaris* transgenic roots.

### *PvRbohA* Downregulation Impairs Rhizobial Infection and IT Progression

To gain insight into the role of *RbohA* during legume root nodule symbiosis, transgenic roots expressing the RNAi and control constructs were inoculated with *R. tropici* expressing a GUS marker (Vinuesa et al., 2003), and rhizobial infection and nodulation were analyzed under the light microscope. Our observations at 7 dpi revealed typical root hair curling of *Rhizobium*-infected root hair cells in both control and *PvRbohA*-RNAi plants (**Figures 4C,G**). By contrast, IT progression was affected, and ITs were denser and thicker within root transgenic hair cells expressing the *PvRbohA*-RNAi construct than in controls (**Figure 4G**). Most ITs in control cells were branched and penetrated the dividing cortical cells, whereas the ITs in *PvRbohA*-RNAi plants failed to reach the dividing cortical cells. The subsequent stages of growth, development, and rhizobial colonization of primordia and young and mature nodules were normal in control roots (**Figures 4D–F**). The mature control nodules show a typical central rhizobial infection zone surrounded by the cortex (**Figure 4F**). By contrast, all stages of nodule development in the *PvRbohA*-RNAi plants display a strong IT disruption phenotype, and rhizobial colonization was blocked in primordia and young and mature nodules (**Figures 4H–J**). The ITs in *PvRbohA*-RNAi plants occasionally spread beyond the root hair cells into dividing cortical cells of primordia or young nodules (**Figure 4J**). The IT progression in root hair cells and dividing cortical cells was quantitatively determined, and found to be significantly reduced at 7 dpi in *PvRbohA*-RNAi plants compared with that of controls (**Figure 4K**). The number of IT abortions per root was remarkably greater in *PvRbohA*-RNAi roots compared to controls transgenic roots at all days including 5, 7, and 9 dpi (**Figure 4L**). These combined data suggest that *RbohA* is required for successful rhizobial penetration, IT progression, and rhizobial colonization.

The production of ROS is correlated with expression of the early nodulin gene *RIP1* in *M. truncatula* (Cook et al., 1995; Ramu et al., 2002). Transcriptional activation of *ERN1* (Middleton et al., 2007; Cerri et al., 2017; Kawaharada et al., 2017; Yano et al., 2017) and *NIN* (Madsen et al., 2010) regulate the early steps of nodulation, such as NF-induced gene expression and IT formation, whereas *ENOD40* was upregulated during cortical cell division and nodule development (Stougaard, 2000). To determine whether the reduced numbers of ITs are associated with changes in the expression of genes involved in early nodulin signaling, we performed RT-qPCR analysis of the transcript levels of *PvRIP1*, *PvERN1*, *PvENOD40*, and *PvNIN* in *Rhizobium*-inoculated transgenic roots (**Figure 4M**). *ENOD40* transcript levels were upregulated, whereas the relative expression levels of all other analyzed genes were unchanged in rhizobial-inoculated *PvRbohA*-RNAi plants. The suppressed expression levels of early nodulin genes in *PvRbohA*-RNAi roots correlated with the reduced numbers of ITs in root hairs and dividing cortical cells (**Figures 4B–M**).

### *PvRbohA* Downregulation Reduces ROS Production

**FIGURE 5:**
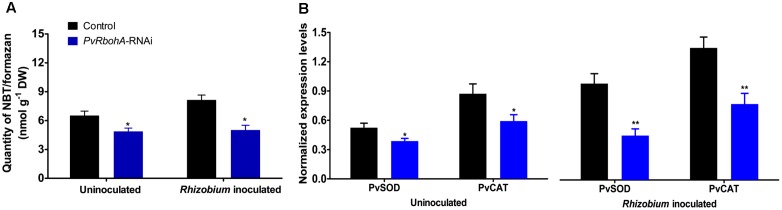
Superoxide accumulation in *Rhizobium*-inoculated transgenic roots. Uninoculated and *R. tropici*-inoculated (72 hpi) transgenic hairy roots were used to estimate superoxide accumulation. **(A)** Quantity of NBT/formazan precipitation in uninoculated and *Rhizobium*-inoculated *PvRbohA*-RNAi roots were biochemically estimated. **(B)** Quantitative RT-PCR analysis of *PvSOD* and *PvCAT* in transgenic *PvRbohA*-RNAi and control roots. Transcript accumulation was normalized to the expression of the *EF1*α and *IDE* reference genes. Values represent the averages of three biological replicates (*n* > 9). The statistical significance of differences between uninoculated and *R. tropici* inoculated samples was determined using an unpaired two-tailed Student’s *t*-test (^∗^*P* < 0.05; ^∗∗^*P* < 0.01). Error bars represent means ± SEM.

Next, we evaluated ROS production in uninoculated and *R. tropici*-inoculated *PvRbohA*-RNAi roots. Uninoculated and *R. tropici*-inoculated [72 hpi (hours post-inoculation)] transgenic roots were stained with NBT to detect superoxide, and the superoxide concentrations were biochemically quantified (Ramel et al., 2009). Compared with the controls, the *PvRbohA*-RNAi roots showed significantly lower levels of superoxide accumulation in uninoculated roots; even after *R. tropici* inoculation, the superoxide levels were not induced (**Figure 5A**). We performed RT-qPCR analysis to measure the transcript levels of superoxide dismutase (*SOD*) and catalase (*CAT*), which are involved in cellular redox homeostasis and regulate cellular ROS concentrations (Rubio et al., 2007), in uninoculated and *R. tropici*-inoculated *PvRbohA*-RNAi. The expression levels of *PvSOD* and *PvCAT* were reduced significantly in uninoculated and *Rhizobium*-inoculated roots of *PvRbohA*-RNAi plants compared with the levels in control roots (**Figure 5B**). In control roots, *Rhizobium* inoculation induced increases in the transcript levels of *PvSOD* and *PvCAT* (**Figure 5B**; Arthikala et al., 2014). These results indicate that *PvRbohA* downregulation reduced superoxide production and attenuated the expression of antioxidant genes in transgenic roots.

### *PvRbohA* Downregulation Impairs Nitrogen Fixation and Disrupts Nodule Morphology

**FIGURE 6:**
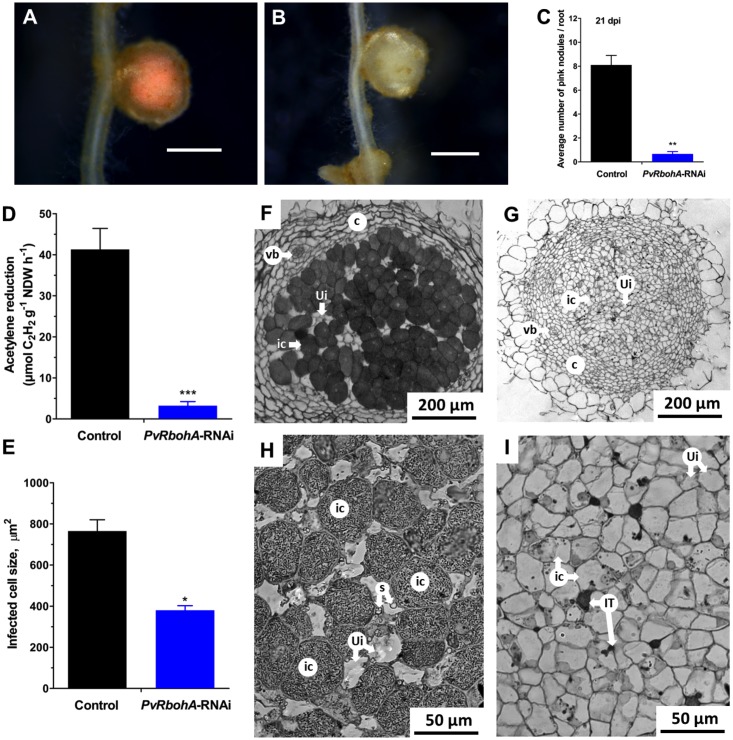
Qualitative and quantitative analysis of nodules generated in transgenic roots. Representative images of 21-day-old *P. vulgaris* transgenic nodules colonized with *R. tropici* and analyzed by bright-field stereomicroscopy. **(A)** Pink nodules on the transgenic control roots. **(B)** Nodules on *PvRbohA*-RNAi roots were pale and white. Bars: **(A,B)** 2 mm. **(C)** Quantitative analysis showing the average number of pink nodules on transgenic control and *PvRbohA*-RNAi roots at 21 dpi. Values represent the averages of three biological replicates (*n* > 27). The statistical significance of differences between control and *PvRbohA*-RNAi root samples was determined using an unpaired two-tailed Student’s *t*-test (^∗∗^*P* < 0.01). Error bars represent means ± SEM. **(D)** Nitrogenase activity was determined by an acetylene reduction assay in transgenic control and *PvRbohA*-RNAi nodules. **(E)** Diagram shows the infected cell size of nodules from transgenic control and *PvRbohA*-RNAi roots. Values represent the averages of three biological replicates [for **(D,E)**, *n* > 27]. The statistical significance of differences between data from transgenic control and *PvRbohA*-RNAi nodules was determined using an unpaired two-tailed Student’s *t*-test (^∗^*P* < 0.05; ^∗∗∗^*P* < 0.001). Toluidine blue-stained transverse sections of a *R. tropici*-inoculated nodule at 21 dpi shows the morphology and organization of representative samples collected from transgenic control **(F)** and *PvRbohA*-RNAi **(G)** roots. Higher magnification images show infected and uninfected cells in nodules from transgenic control **(H)** and *PvRbohA*-RNAi roots **(I)**. vb, vascular bundle; c, cortex; ic, infected cell; ui, uninfected cell; s, starch granules; IT, infection thread.

The combined results indicate that *PvRbohA* downregulation affects rhizobial invasion in transgenic root hairs, and most ITs in these plants failed to reach the dividing cortical cells (**Figures 4G–I**). To further determine the effect of *RbohA* downregulation, we assessed the nodules developed at 21 dpi in transgenic roots colonized by *R. tropici*. The total number of nodules and the fresh weight of nodules and shoots decreased by 62, 68, and 30%, respectively, in *PvRbohA*-RNAi roots compared with control roots (Supplementary Figure S4). Stereomicroscopy observations confirmed that nodules on control roots were pink (**Figure 6A**), which is indicative of leghemoglobin expression and nitrogenase activity (Ott et al., 2005), whereas 92% of nodules on *PvRbohA*-RNAi roots at 21 dpi were pale (**Figures 6B,C**). Next, we quantified the nitrogen-fixing capacity of the sparse transgenic nodules by performing an acetylene reduction assay. The nitrogen-fixing capacity of the *PvRbohA*-RNAi lines was 92% lower than that of controls (**Figure 6D**), indicating that the *PvRbohA*-RNAi nodules were defective in nitrogen fixation. The transcript levels of NADH-dependent glutamate synthase II (GOGAT), a key enzyme in primary ammonia assimilation in *P. vulgaris* nodules (Blanco et al., 2008), were also significantly lower in *PvRbohA*-RNAi nodules than in controls (Supplementary Figure S5).

We then used histological analyses to examine the structural characteristics of *PvRbohA*-RNAi nodules. Nodule sections from both control and *PvRbohA*-RNAi lines display similar organization, including an outer cortex encircled by an inner cortex containing the nodule vascular bundles and the central tissue (**Figures 6F,G**). However, the central tissue of *PvRbohA*-RNAi nodules was only weakly stained with toluidine blue, indicating fewer symbiosomes in infected cells, and did not show any significant increase in cell size (**Figure 6G**). By contrast, symbiosomes were densely packed in the infected cells of controls (**Figure 6F**). The sizes of infected cells of *PvRbohA*-RNAi nodules remained the same as those of uninfected cells. The infected cell size of *PvRbohA*-RNAi nodules was 380 ± 22 μm^2^, relative to 765 ± 55 μm^2^ in controls (**Figure 6E**). Starch granules were absent from *PvRbohA*-RNAi nodules compared with the control nodules, which show abundant starch granules (**Figures 6H,I**). In contrast to indeterminate nodules from IRLC legumes, in which bacteroids are terminally differentiated and unable to restore their division capacity (Montiel et al., 2016b), in *P. vulgaris* nodules, bacteroids maintain their ability to reproduce on agar plates. We determined the number of rhizobia present in control and *PvRbohA*-RNAi nodules by CFU assays, and found that the number of rhizobia declined by 78% in *PvRbohA*-RNAi nodules compared with controls (Supplementary Figure S6). This observation supports our earlier histological observations of nodules in *PvRbohA*-RNAi lines and controls (**Figures 6G,I**).

### *RbohA* Downregulation Affects the Expression of AUX1, Cyclins, and Polyamines

**FIGURE 7:**
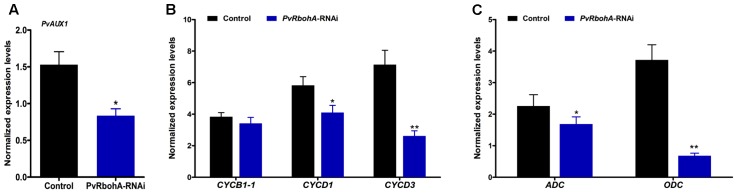
Transcript accumulation pattern of AUX1, cyclins, and polyamine synthesis genes in rhizobia-infected *PvRbohA*-RNAi roots at 7 dpi. **(A)** Transcript levels (normalized to uninoculated roots) of *P. vulgaris AUX1*, **(B)** *CYCB1-1*, *CYCD1*, and *CYCD3* cyclin genes, and **(C)** *ADC* and *ODC* polyamine synthesis genes in control and *PvRbohA*-RNAi roots. Transcript accumulation was normalized to the expression of the *EF1*α and *IDE* reference genes. Values represent the averages of three biological replicates (*n* > 9). The statistical significance of differences between control and *PvRbohA*-RNAi samples was determined using an unpaired two-tailed Student’s *t*-test (^∗^*P* < 0.05; ^∗∗^*P* < 0.01). Error bars represent means ± SEM. dpi, days post-inoculation; ADC, arginine decarboxylase; ODC, ornithine decarboxylase.

*PvRbohA* downregulation induced several defects in the developmental program of *P. vulgaris* nodules, leading to reduced sizes of infected cells. This prompted us to explore the expression of genes linked to ROS metabolism and nodule organogenesis in *PvRbohA*-RNAi plants inoculated with rhizobia at 7 dpi. RBOH-mediated ROS function is an important signal for auxin-regulated cell division during lateral root formation (Orman-Ligeza et al., 2016), and homologs of the auxin transporter AUX1-like genes are expressed during nodule primordia development in *M. truncatula* (de Billy et al., 2001). RT-qPCR analysis revealed that *PvAux1* was slightly induced after rhizobial infection compared with non-inoculated roots of the same age; however, this response was abolished in *PvRbohA*-RNAi roots (**Figure 7A**). Auxin has a crucial role in stimulating the cell cycle by shortening the G_1_ phase (Perrot-Rechenmann, 2010). Transcript levels of the G_1_ cell-cycle genes *CYCB1-1*, *CYCD1*, and *CYCD3* were dramatically increased at 7 dpi in control transgenic roots (**Figure 7B**). Similarly, *PvRbohA*-RNAi roots showed clear increases in the expression of these cyclin genes, but to a lesser extent than observed in non-silenced transgenic roots (**Figure 7B**). As cellular polyamine is known to regulate cell growth (Wang et al., 2003), and the expression levels of genes involved in their synthesis are induced at early stages of nodulation (Efrose et al., 2008), we measured the transcript levels of key enzymes involved in polyamine biosynthesis, such as arginine decarboxylase (ADC) and ornithine decarboxylase (ODC), which participate in alternative polyamine biosynthesis pathways (Malmberg et al., 1998). The expression levels of *ADC* and *ODC* genes were significantly induced after rhizobial infection at 7 dpi (**Figure 7C**). In *PvRbohA*-RNAi roots, *ADC* transcript levels were slightly increased but *ODC* transcript levels were moderately downregulated in response to rhizobial inoculation at 7 dpi (**Figure 7C**). These results support the observed defects in nodule organogenesis in RNAi lines, indicating that *RbohA* is crucial for nodule development in *P. vulgaris*.

## Discussion

*Rboh*s constitute important gene families in many plant species. Redundant and non-redundant roles have been described for specific *Rboh*s that are involved in the same biological process (Torres et al., 2002; Kwak et al., 2003; Zhang et al., 2009; Lassig et al., 2014; Morales et al., 2016; Liu et al., 2017). Our group showed that silencing of *PvRbohB* impairs rhizobial infection and nodule organogenesis in the *P. vulgaris*–*Rhizobium* symbiosis (Montiel et al., 2012). Here, we examined *PvRbohA*, which is a highly abundant *Rboh* transcript in *P. vulgaris* nodules. The results indicate that *PvRbohA* and *PvRbohB* share similar functions during IT progression and development of nodule primordia, although colonization of root hairs and nodule cells seems to be preferentially controlled by *PvRbohA*. This work shows that specific *Rboh*s have crucial roles in the legume–*Rhizobium* symbiosis.

### Role of PvRBOHA during Rhizobial Invasion and IT Progression

The first *Rboh* gene was identified in *Oryza sativa*, and further analyses showed that these genes are widespread in plants and belong to gene families of variables sizes (Groom et al., 1996; Torres et al., 1998; Montiel et al., 2012; Chang et al., 2016). Despite these large gene families, some *Rboh*s appear to have specialized roles, such as *AtRbohC* and *AtRbohH*/*AtRbohJ*, which function in Arabidopsis root hair growth and pollen tube growth, respectively (Foreman et al., 2003; Takeda et al., 2008; Lassig et al., 2014). By contrast, *AtRbohD* and *AtRbohF* act redundantly and/or synergistically during stomatal closure and following exposure to certain pathogens or oxygen deficiency (Torres et al., 2002; Kwak et al., 2003; Liu et al., 2017). Similarly, the present and previous reports (Montiel et al., 2012; Arthikala et al., 2014) indicate that both *PvRbohB* and *PvRbohA* are required for IT progression and nodule development in the *P. vulgaris*–*Rhizobium* symbiosis (**Figures 4G–J**). The promoters of these genes are activated in the root hair cells harboring ITs (**Figures 3C–E**); however, their subcellular distributions show notable differences. PvRBOHA seems to be homogenously localized at the plasma membrane of root hairs (**Figure 2G**), whereas PvRBOHB is localized in the central apical dome (Montiel et al., 2012). During rhizobial infection, both oxidases are localized in the infection pocket; however, while PvRBOHB was observed in the migration point of the IT, PvRBOHA was detected throughout the IT. Different subcellular distributions of RBOHs in root hairs led to two models that explain root hair development; RBOH-mediated ROS production in the root hair tip is required to maintain apical growth, whereas ROS in the flanking regions mediates cell wall extensibility (Foreman et al., 2003; Knight, 2007; Monshausen et al., 2007). *PvRbohA* silencing reduces the number of infection events (root hairs invaded by rhizobia; **Figure 4K**), whereas *PvRbohB* downregulation produces a similar ratio of infection events *per* root as that in control transgenic roots (Montiel et al., 2012). We propose that rhizobial invasion in *P. vulgaris* is a coordinated process that involves the participation of at least two RBOHs (PvRBOHB and PvRBOHA) to sustain IT progression. These oxidases likely modulate cell wall flexibility through an interplay with calcium signaling, vesicle trafficking, small GTPases, cell wall proteins, and cytoskeleton components (Zepeda et al., 2014; Montiel et al., 2016a).

### Specific and Overlapping Expression Profiles of *PvRboh*s during Nodulation

Although some *Rboh*s display similar expression profiles, most of these genes display distinct expression patterns (Marino et al., 2011; Montiel et al., 2012, 2016a; Belmondo et al., 2016; Chang et al., 2016; Kaur and Pati, 2016; Morales et al., 2016). This is largely due to the arrangements of diverse motifs in their promoter regions, ranging from stress-responsive to developmental elements (Kaur and Pati, 2016). The promoter activities of *MtRbohA* and *MtRbohB* in *M. truncatula* indeterminate nodules are detected primarily in the nitrogen-fixing zone and zone I–III, respectively. By contrast, *MtRbohE*/*MtRbohF* and *MtRbohG* promoters are preferentially active in the vascular tissue and meristematic region of *M. truncatula* nodules, respectively (Marino et al., 2011). These data are consistent with the observed transcript levels of these genes in different zones of *M. truncatula* nodules as determined by RNAseq (Roux et al., 2014; Montiel et al., 2016a). In *P. vulgaris*, both *PvRbohA* and *PvRbohB* promoters were induced during nodule development (**Figures 3F–H**); however, the *PvRbohA* promoter activity was limited primarily to vascular bundles in the emerging nodule primordia and in nitrogen-fixing nodules (**Figures 3G,I**). *PvRbohA* expression was not associated with infected cells in active nodules, and was higher in the central tissue of senescent nodules (**Figures 3J,K**). This observation is consistent with the high levels of *PvRbohA* transcripts observed during nodule senescence (Supplementary Figure S1). None of the analyzed *MtRboh*s are induced during nodule senescence in *M. truncatula* (Marino et al., 2011).

The putative orthologous genes *MtRboh* and *PvRboh* display remarkably different expression profiles in tissues and organs of *M. truncatula* and *P. vulgaris*, respectively (Marino et al., 2011; Montiel et al., 2012, 2016a). Phylogenetic analysis of various RBOHs indicated that *PvRbohA* is highly homologous to *MtRbohE*/3 (Montiel et al., 2012), whose promoter is active in the apical region of *M. truncatula* nodules; however, silencing *MtRbohE* does not affect nodule formation (Belmondo et al., 2016). Downregulation of *MtRbohA* affects nitrogen fixation in *M. truncatula* nodules, but the initial stages of the symbiotic process are not apparently affected (Marino et al., 2011). These differences could be attributed to the distinct developmental programs in indeterminate and determinate nodules, although they may also be due to evolutionary differences among *Rboh* family members. Similarly, the putative orthologous genes *OsRboh* and *AtRboh* display clear differences in their expression patterns in the same organs (Chang et al., 2016). The only known *Rboh* mutation that affects root hair growth is *rbohC* in *A. thaliana* (Foreman et al., 2003; Takeda et al., 2008). In *P. vulgaris*, root hair development is not affected in *PvRbohA*-silenced or *PvRbohB*-silenced plants, presumably due to compensation from other *Rboh* members. A recent report showed that Arabidopsis RBOH function depends on other traits in addition to proper tissue-specific expression. The typical AtRBOHD-dependent ROS production after *Plectosphaerella cucumerina* treatment, which was abolished in the *rbohD* mutant, was not restored in this mutant background expressing *AtRbohF* under the transcriptional regulation of the *AtRbohD* promoter, but only in plants expressing *AtRbohD* under the control of its cognate promoter (Morales et al., 2016). This study illustrates the relevance of other regulatory elements in these oxidases. In particular, the N-terminus region of these proteins is a target for regulation by calcium, phosphorylation, and small GTPase-binding proteins (Kaur et al., 2014). Silencing of specific small GTPases has an adverse effect on nodule formation in *Glycine max* and *M. truncatula* (Ke et al., 2012; Kiirika et al., 2012; Lei et al., 2015). However, a direct connection with *Rboh* genes was only explored for MtROP9 (Kiirika et al., 2012).

### *PvRbohA* Silencing Induces Misregulation of Several Genes Involved in Nodule Organogenesis

The expression of genes encoding antioxidant enzymes and proteins related to cell division and auxin metabolism was significantly disrupted in *PvRbohA*-RNAi roots (**Figures 5B**, **7**). Rhizobial infection triggers superoxide accumulation at 72 hpi (**Figure 5A**), which correlates with the onset of nodule primordium formation, a process with high meristematic activity (Ramu et al., 2002; Montiel et al., 2016a). This increase in ROS accumulation is accompanied by upregulation of the ROS-scavenging genes *SOD* and *CAT*, and upregulation of *PvAux1* and *ODC* involved in auxin homeostasis and cell growth, respectively (Wang et al., 2003; Benjamins and Scheres, 2008; Efrose et al., 2008). However, both the increase in ROS levels and the upregulation of antioxidant genes are suppressed in *PvRbohA*-RNAi roots at this time point (**Figure 5A**). This latter observation suggests that this oxidative response is required for a successful symbiotic process, based on the defects shown in *PvRbohA*-silenced roots. This notion is further reinforced, since ROS have been linked to cell proliferation, and recent work supports the role of RBOH during lateral root formation through a signaling pathway involving auxins (Tsukagoshi et al., 2010; Orman-Ligeza et al., 2016). The expression pattern of the *PvRbohA* promoter (**Figures 3F–H**) was similar to that of AUX1-like genes in *M. truncatula* during nodule primordium formation. Transcripts of these auxin transporters were detected in cells that were likely derived from the pericycle and later associated with the vascular bundles (de Billy et al., 2001). Proper nodule organogenesis requires reactivation of mitotic activity in cortical cells, where cyclins promote meristem formation (Roudier et al., 2003; Vinardell et al., 2003). The accumulation of *CYCB-1*, *CYCD1*, and *CYCD3* transcripts was higher in *PvRbohA*-RNAi roots than in uninfected roots, although the levels were lower than those in inoculated control transgenic roots (**Figure 7B**). Similarly, the transcript levels of *ADC* and *ODC* genes required for polyamine synthesis were affected in rhizobia-inoculated *PvRbohA*-RNAi roots (**Figure 7C**). The progressive accumulation of polyamines during maturation of *Lotus japonicus* nodules suggests a role for polyamines during nodule maturation (Efrose et al., 2008). The moderate expression of genes required for cell division and growth may explain the formation of few and small nodules in *PvRbohA*-RNAi roots (**Figure 6C** and Supplementary Figure S4).

### Role of *PvRbohA* and Its Interplay with Other Actors in the Nodulation Process

The data collected in this study show that loss-of-function of *PvRbohA* affects different stages of the *P. vulgaris–R. tropici* symbiosis. The reduced infection events in *PvRbohA*-RNAi plants could be due to two non-exclusive reasons. In the first scenario, PvRBOHA contributes to rapid and transient ROS production in response to NF perception in the root hairs (Cárdenas et al., 2008). This oxidative burst is likely part of the nodulation signaling pathway, necessary for downstream processes that precede rhizobial colonization. This notion is further supported by the reduced expression of the transcription factors ERN1 and NIN in the *PvRbohA*-RNAi lines (**Figure 4M**). The second possibility is that this oxidase facilitates cell wall modifications in the root hair tip, to trap the rhizobia in the infection pocket. This latter phenomenon seems to be coordinated with PvRBOHB activity, since both proteins were visualized in the infection pockets (Montiel et al., 2012; Supplementary Figures S2C,D). In addition, PvRBOHA likely has a prominent role at the base of the root hair, which would explain the abortion of ITs in these cells. This hypothesis is further reinforced by the localization of this oxidase during IT progression in the root hairs. The crosstalk between the base of the root hairs containing ITs and the adjacent cortex cells has been poorly explored in nodulation; however, it is known that pre-ITs mark the course of IT progression in the outer cortex cells (Brewin, 2004). Interestingly, the YFP-PvRBOHA chimera was observed only at one pole of the cortex cells, adjacent to the infection site, and was not homogenously distributed throughout these cells. The promoter activity of *PvRbohA* indicates that this gene is not expressed in the cortical cells of nodule primordia at 5 dpi, but rather in the cells that give rise to the vascular bundles (**Figure 3G**). As previously discussed, the expression in the vascular tissue is similar to that reported for several AUX-like genes during nodule organogenesis of *M. truncatula* (de Billy et al., 2001). This current study reinforces the connection between RBOHs and auxins, suggesting that a circuit connecting ROS and auxins promotes nodule development. Even though the promoter region of *PvRbohA* was not active in the infected nodule cells, this oxidase likely participates in the release of rhizobia from ITs, since rhizobial colonization in nodule cells was drastically reduced in *PvRbohA*-RNAi nodules (**Figure 6I**).

Our group recently reviewed the versatile functions of *Rboh*s at different steps in legume–rhizobia symbioses (Montiel et al., 2016a). The current study further confirms the central contribution of different *Rboh* members to the establishment of this mutualistic association, and supports the characterization of other *Rboh* genes to fully understand the coordination of these oxidases with other nodulation genes.

## Author Contributions

M-KA conducted the experiments, analyzed the data, and wrote the article. JM isolated and cloned the *PvRbohA* gene, made the RNAi construct, conceived the study, and contributed in drafting manuscript. RS-L assisted with histology and microscopy. NN generated transgenic hairy roots and assisted in inoculation experiments with rhizobia. LC critically evaluated the data. CQ conceived and coordinated the study and finalized the article. All authors read and approved the final article.

## Conflict of Interest Statement

The authors declare that the research was conducted in the absence of any commercial or financial relationships that could be construed as a potential conflict of interest.
