# Supplementary material for: Respiratory Burst Oxidase Homolog Gene A Is Crucial for Rhizobium Infection and Nodule Maturation and Function in Common Bean
Source: Front Plant Sci. 2017 Nov 23;8:2003. doi: 10.3389/fpls.2017.02003 (PMC5703732; doi:10.3389/fpls.2017.02003)
Supplement: Supplementary file 1 [file Table_1.DOCX]

Supplementary Material

Respiratory burst oxidase homolog gene A is crucial for *Rhizobium* infection and nodule maturation and function in common bean

Manoj-Kumar Arthikala, Jesús Montiel, Rosana Sánchez-López, Noreide Nava Luis Cárdenas, and Carmen Quinto*

*** Correspondence:** Dr. Carmen Quinto: [quinto@ibt.unam.mx](mailto:quinto@ibt.unam.mx)

**Supplementary Table S1 |** Primer sequences of *Phaseolus vulgaris* genes used for cloning and RT-qPCR.

| Gene (IDs) | | Oligonucleotide sequence |
| --- | --- | --- |
| *pPvRbohA*  (Phvulv091020621) | F | 5′-TTC ACC AAC GAA GGT CCA TTG AG-3′ |
|  | R | 5′-TTT CTG ATG AAC ACA CAG TGA CTC ACA C-3′ |
| YFP-*PvRbohA*  (Phvulv091020621) | F | 5′-ATG GGA GGA GCT TCT GCA G-3′ |
|  | R | 5′-CAA CCA CCT CTT GCC TTC ATC-3′ |
| *PvRbohA*-RNAi  (Phvulv091020621) | F | 5′-CAC CTG ACA GGA TAA GAA GTG AAG G -3′ |
|  | R | 5′-GAC ACT GAG ACA CAT CCA AGC-3′ |
| *PvRbohA*  (Phvulv091020621) | F | 5′-GTG GAC TTC TAA GAG CAG AGT G-3′ |
|  | R | 5′-CAG CAG AAC CAC CTC GTA CTC-3′ |
| *PvRbohB*  (Phvulv091013731) | F | 5′-GGA AGG AGA TGC TCG ATC TGC-3′ |
|  | R | 5′-GTC TTC ACC CTT GTC CCT GAA AC-3′ |
| *PvRbohC*  (Phvulv091002467) | F | 5′-CCG GAG TCT CAA AGT CAA GTT C-3′ |
|  | R | 5′-GAG CAC TGG GAC TTC CAT CTC-3′ |
| *PvRbohD*  (Phvulv091005473) | F | 5′-CTA TAC GGA TGC GAA AGG C-3′ |
|  | R | 5′-CAG TGC TAG AAC ATT TCC TGG G-3′ |
| *PvRbohE*  (Phvulv091006195) | F | 5′-CAG CAC AAG ACT ACC AGC GC-3′ |
|  | R | 5′-CCT CCT GGT GTT AAG TTT GAC G-3′ |
| *PvRbohF*  (Phvulv091020738) | F | 5′-CTA GTC GGT CTC GGG ATA GGA G-3′ |
|  | R | 5′-GAG AGA TGG TGA GTC AGT GG-3′ |
| *PvRbohG*  (Phvulv091007712) | F | 5′-GAA CAC TCG GAG ACT GGA GC-3′ |
|  | R | 5′-GGA CAT CCC TGT GAT CGT G-3′ |
| *PvRbohH*  (Phvulv091002934) | F | 5′-GAT GTA GTT TCA GAA AGC CGG-3′ |
|  | R | 5′-CTA TCC GAG AAC TTT GAT GCG AG-3′ |
| *PvRbohI*  (Phvulv091023888) | F | 5′-GTC TCC GAT TCA AGT AGA GG-3′ |
|  | R | 5′-GAA CCT TGT TCC CTT GTC AC-3′ |
| *PvEf1α*  (Phvul.004G075100) | F | 5′-GGT CAT TGG TCA TGT CGA CTC TGG-3′ |
|  | R | 5′-GCA CCC AGG CAT ACT TGA ATG ACC-3′ |
| *PvIDE*  (Phvul.001G133200) | F | 5′-GCA ACC AAC CTT TCA TCA GC-3′ |
|  | R | 5′-AGA AAT GCC TCA ACC CTT TG-3′ |
| *PvSOD*  *(CuZnSOD)*  (Phvul.006G097000) | F | 5′- TAC TGG AAA TGC TGG TGG C-3′ |
|  | R | 5′- GAC CGA ACC TAA CCA AAC GGG-3′ |
| *PvCAT*  (Phvul.007G135400) | F | 5′-CAC ATC CAG GAG AAT TGG AGG-3′ |
|  | R | 5′-CCA GCT TTG CTG ATG AGG GTG-3′ |
| *PvRIP1*  (Phvul.001G111800) | F | 5′-GTC GAA TCT CGC CTT GT-3′ |
|  | R | 5′-GGC CCT GTT GTA TCT TGT GC-3′ |
| *PvERN1*  (Phvul.001G111800) | F | 5′-GGA GCT GTC TTT GAT CGT TTT CC-3′ |
|  | R | 5′-CAA ATT CAG AAA GCT CCA AGT CAG C-3′ |
| *PvENOD40*  (Phvul.002G064200) | F | 5′-AGT TTT GTT GGC AAG CAT CC-3′ |
|  | R | 5′-TAA GCA CAA GCA AAC TGT TG-3′ |
| *PvNIN*  (Phvul.009G115800) | F | 5′-GGG GAT TCA GAG ATT TGC AG-3′ |
|  | R | 5′-AAC CCA CTC TTG AGC ATC GT-3′ |
| *PvAUX1*  (Phvul.008G225300) | F | 5′-GAA GTG CTG GAA GGT TTA CTA GGT C-3′ |
|  | R | 5′-CTC CGA ATA TGT AAG TCC AGG TCC-3′ |
| *PvCycD3*  (Phvul.007G038400) | F | 5′-GAC TTG TTC TGG GAA GAC GAG GAA C-3′ |
|  | R | 5′-GGT TCA AAT GCA CAT GAT TGT TAT TA-3′ |
| *PvCycD1*  (Phvul.002G074800) | F | 5′-GCT CCA ACT GCC TCC CAG ATT C-3′ |
|  | R | 5′-ATC CGG CGA TGG AAT CCT CCT C-3′ |
| *PvCycB1*  (Phvul.008G203300) | F | 5′-AGT GTT GTC AAG TGC TTT GCT GGA G-3′ |
|  | R | 5′-GGA TTG CGC CAA AAA CCT AGT-3′ |
| *PvADC*  (Phvul.009G002500) | F | 5′-GAC AGG ATG CCT TAT CTG GTT C-3′ |
|  | R | 5′-CCC ATT TCA AGC AAA CAC GAG C-3′ |
| *PvODC*  (Phvul.009G020500) | F | 5′-GCT GAA GAT GGG CGT TTC TT-3′ |
|  | R | 5′-CAT CTA TGA GTA GGT GTC TCC-3′ |
| *PvGOGAT*  (Phvul.009G053900) | F | 5′-ATG TCT TCA TCA ATC TCG TTT CCC ACG-3′ |
|  | R | 5′-ACG CAA CCG TGT TCC CAA AAA CTT TGT C-3′ |
