# Supplementary material for: Respiratory Burst Oxidase Homolog Gene A Is Crucial for Rhizobium Infection and Nodule Maturation and Function in Common Bean
Source: Front Plant Sci. 2017 Nov 23;8:2003. doi: 10.3389/fpls.2017.02003 (PMC5703732; doi:10.3389/fpls.2017.02003)
Supplement: Supplementary file 2 [file Image_1.PDF]

Supplementary Material

Respiratory burst oxidase homolog gene A is crucial for *Rhizobium* infection and nodule maturation and function in common bean

Manoj-Kumar Arthikala, Jesús Montiel, Rosana Sánchez-López, Noreide Nava Luis Cárdenas, and Carmen Quinto*

*** Correspondence:** Dr. Carmen Quinto: quinto@ibt.unam.mx

Supplementary Figure S1

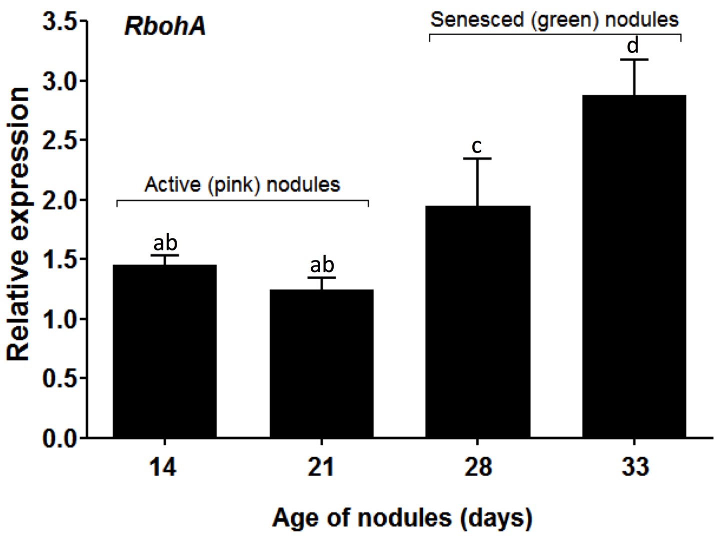

Supplementary Figure S1 | Transcript abundance of *PvRbohA* in wild-type *P. vulgaris* nodules. RT-qPCR analysis of total RNA from 14-, 21-, 28-, and 33-day-old nodules was performed to measure *RbohA* transcript accumulation. Transcript accumulation was normalized to the expression of the *Ef1α* and *IDE* reference genes. Values represent three biological replicates ($n > 9$). The statistical significance of differences between the different timepoints was calculated by ANOVA and Tukey´s Multiple Comparison Test, where different letters mean significance differences ($P < 0.01$). Error bars represent means ± SEM.

Supplementary Figure S2

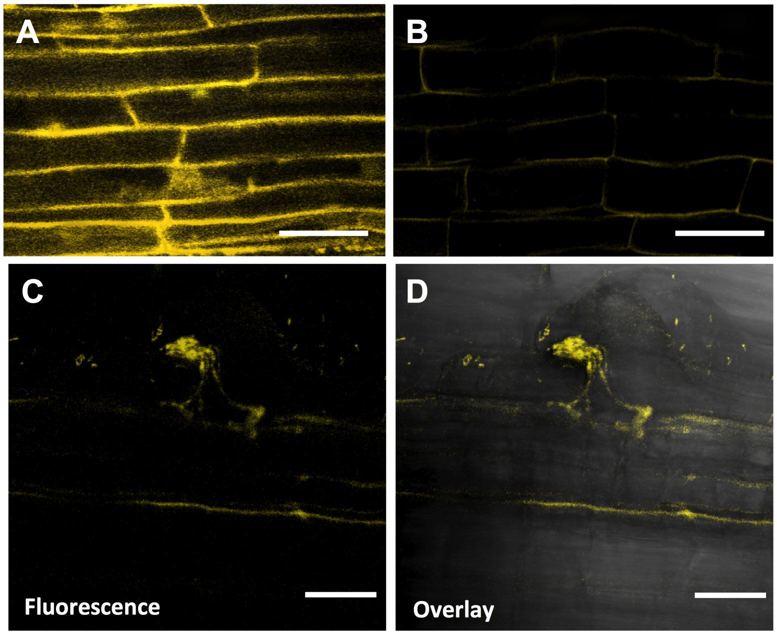

Supplementary Figure S2 | Subcellular localization of PvRBOHA in *P. vulgaris* transgenic roots. The *PvRbohA* ORF was cloned in pEarleyGate104 to construct an N-terminal YFP fusion, and introduced into *P. vulgaris* hairy roots to determine the subcellular localization. Confocal images show YFP fluorescence from transgenic roots expressing **(A)** non-fused 35S-YFP (control) or **(B)** YFP-PvRBOHA in non-inoculated roots and **(C and D)** root hairs infected by rhizobia at 5 dpi with *R. tropici.* Overlay: transmitted light and yellow fluorescence. Bars = 20 µm.

Supplementary Figure S3

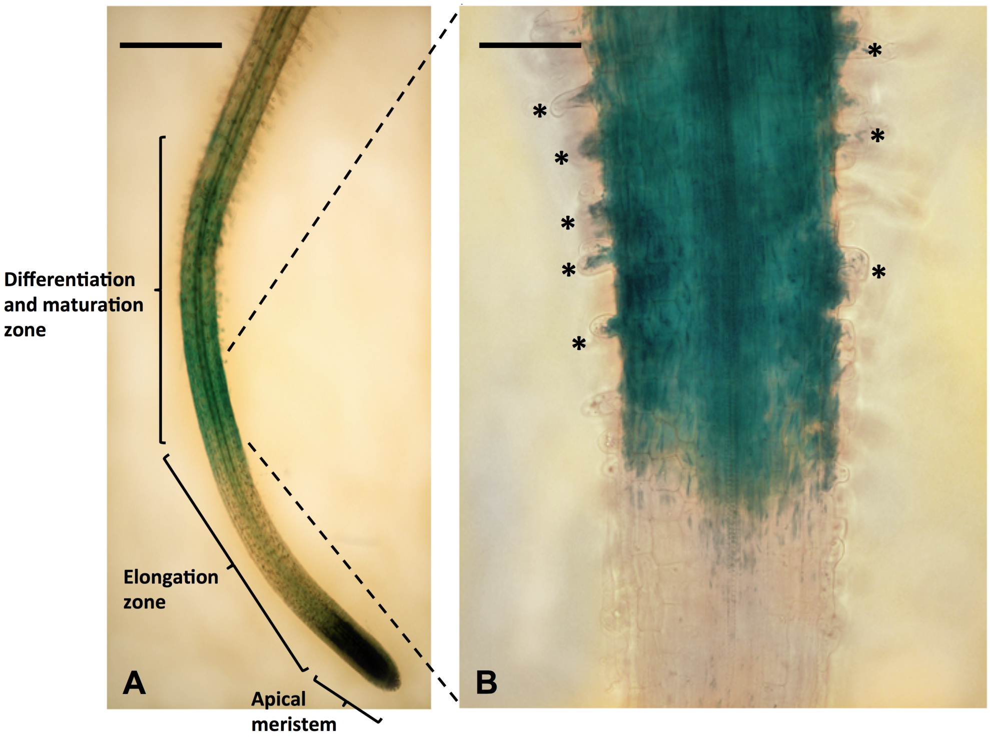

Supplementary Figure S3 | *PvRbohA* promoter analysis in transgenic *P. vulgaris* roots after inoculation with rhizobia. Transgenic roots were inoculated with *R. tropici*, harvested at 72 hpi, and incubated with the GUS substrate. **(A)** GUS staining occurs in the apical meristem and in maturation and differentiation zones. **(B)** Higher magnification of the differentiation zone showing GUS staining in root hair cells (asterisks). Bars: (A) 10 mm, and (B) 200 µm.

Supplementary Figure S4

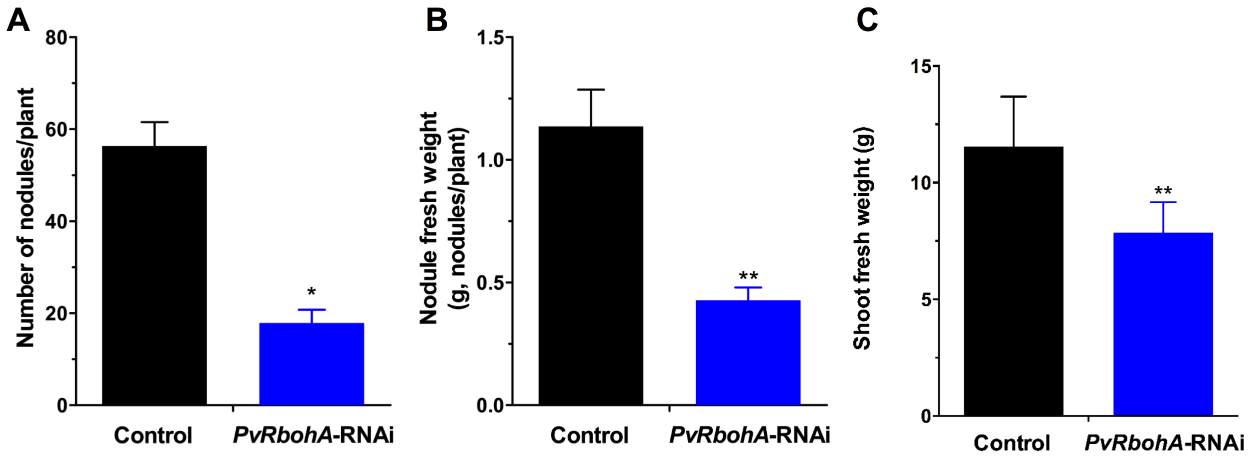

Supplementary Figure S4 | Quantitative analysis of the numbers and weights of transgenic nodules and shoots. (A) Total number of nodules from transgenic control and *PvRbohA*-RNAi roots at 21 dpi. **(B)** Total fresh weight of nodules and **(C)** shoots in transgenic control and *PvRbohA*-RNAi plants at 21 dpi. Values represent the averages of three biological replicates ($n > 27$). The statistical significance of differences between control and *PvRbohA*-RNAi root samples was determined using an unpaired two-tailed Student's *t*-test (*$P < 0.05$; **$P < 0.01$). Error bars represent means ± SEM.

Supplementary Figure S5

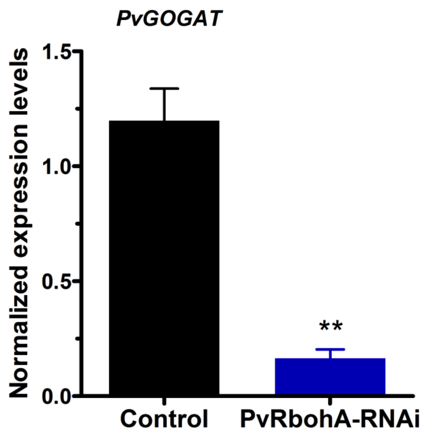

Supplementary Figure S5 | RT-qPCR analysis of *PvGOGAT* expression in transgenic control and *PvRbohA*-RNAi nodules at 21 dpi. Transcript levels were normalized to uninoculated roots. Values represent the averages of three biological replicates ($n > 9$). The statistical significance of differences between control and *PvRbohA*-RNAi samples was determined using an unpaired two-tailed Student's *t*-test (**$P < 0.01$). Error bars represent means ± SEM.

Supplementary Figure S6

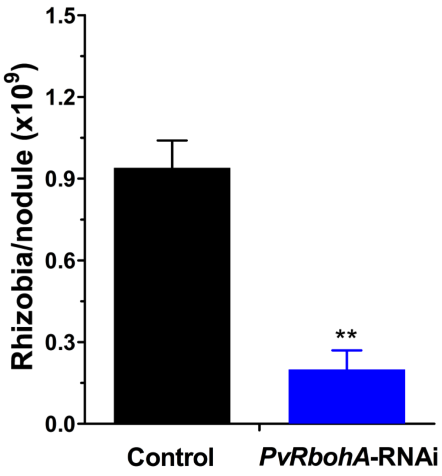

Supplementary Figure S6 | Quantitative analysis of bacterial numbers in transgenic nodules. Number of rhizobial cells isolated from transgenic control and *PvRbohA*-RNAi nodules at 21 dpi. Values represent the averages of three biological replicates ($n > 9$). The statistical significance of differences between transgenic control and *PvRbohA*-RNAi nodules was determined using an unpaired two-tailed Student's t-test (**$P < 0.01$).
